# Kirigami Makes a Soft Magnetic Sheet Crawl

**DOI:** 10.1002/advs.202301895

**Published:** 2023-06-25

**Authors:** Pierre Duhr, Yuki A. Meier, Alireza Damanpack, Julia Carpenter, André R. Studart, Ahmad Rafsanjani, Ahmet F. Demirörs

**Affiliations:** ^1^ Complex Materials Department of Materials ETH Zurich Zurich CH‐8092 Switzerland; ^2^ Department of Mechanical and Electrical Engineering University of Southern Denmark Odense 5230 Denmark; ^3^ SDU Soft Robotics SDU Biorobotics The Maersk Mc‐Kinney Moller Institute University of Southern Denmark Odense 5230 Denmark; ^4^ Present address: Department of Physics University of Fribourg Fribourg CH‐1700 Switzerland

**Keywords:** crawling, kirigami, locomotion, magnetic soft composites, soft robotics

## Abstract

Limbless crawling on land requires breaking symmetry of the friction with the ground and exploiting an actuation mechanism to generate propulsive forces. Here, kirigami cuts are introduced into a soft magnetic sheet that allow to achieve effective crawling of untethered soft robots upon application of a rotating magnetic field. Bidirectional locomotion is achieved under clockwise and counterclockwise rotating magnetic fields with distinct locomotion patterns and crawling speed in forward and backward propulsions. The crawling and deformation profiles of the robot are experimentally characterized and combined with detailed multiphysics numerical simulations to extract locomotion mechanisms in both directions. It is shown that by changing the shape of the cuts and orientation of the magnet the robot can be steered, and if combined with translational motion of the magnet, complex crawling paths are programed. The proposed magnetic kirigami robot offers a simple approach to developing untethered soft robots with programmable motion.

## Graphical abstract

Introducing a simple kirigami pattern into a thin, soft magnetic sheet turns it into a soft crawling robot when subjected to a rotating external magnetic field. The robot can crawl forward and backward depending on the direction of the rotation of the external field and steer in‐plane by rotating the axis of the rotation of the magnet.

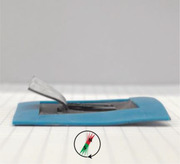

## 1 Introduction

Limbless organisms crawl in myriad patterns by pushing their bodies against various substrates.^[^ ^1^ ^]^ Crawling in many animalsoften relies on synchronizing the body movement with the orchestrated motion of several tiny foot‐like features in contact with the ground. Among vertebrates, snakes crawl in straight lines during rectilinear locomotion by combining axial movements of their body with anisotropic friction of their scaly skin.^[^ ^2^, ^3^, ^4^ ^]^ Limbless invertebrates adapted to other crawling gaits to navigate crannies and traverse complex terrains. For example, earthworms use antagonistic muscle contractions to creep over the ground or burrow through the soil while augmenting their locomotion with small setae that produce directional friction.^[^ ^5^, ^6^ ^]^ Caterpillars crawl by generating traveling waves and controlling the timing of releasing their prolegs' firm grip.^[^ ^7^ ^]^

Limbless locomotion has inspired mobile soft robots that could still achieve complex motions despite their simple and inexpensive fabrication.^[^ ^8^, ^9^ ^]^ Most existing soft robots consist of an unstructured surface with no control over friction,^[^ ^10^, ^11^, ^12^ ^]^ and efforts that combine friction anisotropy with simple design and control strategies are rare.^[^ ^13^, ^14^, ^15^, ^16^ ^]^ As a result, to locomote, robots must activate several independent actuators or require sophisticated external actuation mechanisms. Soft magnetic robots made of magnetoactive elastomers based on ferromagnetic particles have recently shown enormous potential for realizing untethered, entirely soft robots controlled by an external magnetic field.^[^ ^17^ ^]^ A critical limitation of existing continuum magnetic soft robots is their global deformation and lack of localized actuation.^[^ ^18^, ^19^, ^20^, ^21^, ^22^, ^23^, ^24^ ^]^ Distributed actuation of magnetoactive soft robots relies on rigid permanent magnets that compromise the system's compliance.^[^ ^25^, ^26^ ^]^ Therefore, a strategy that can enable tuning the local dynamics of soft robots under a simplified global actuation is required. Here, we adopted kirigami, the art of paper cutting, which has enabled various innovative morphing structures.^[^ ^27^, ^28^, ^29^, ^30^, ^31^, ^32^, ^33^ ^]^ Researchers recently employed magnetic fields for untethered actuation of kirigami‐inspired morphing systems^[^ ^34^, ^35^ ^]^ and soft grippers.^[^ ^36^ ^]^

**Figure 1:**
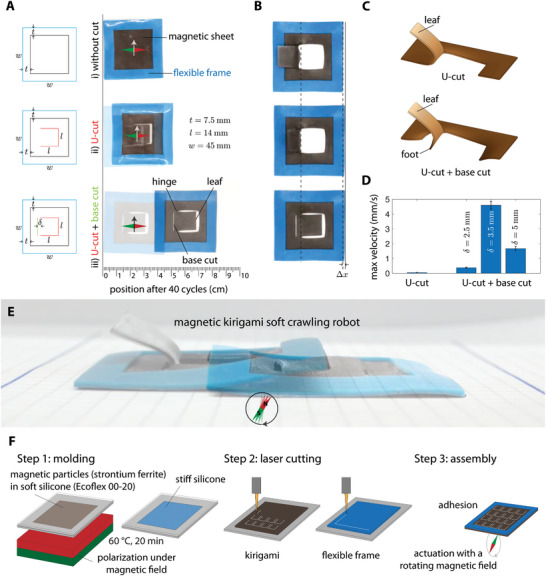
Working principle, design, and fabrication. A) Preliminary experiments showing the effect of geometrical parameters on the locomotion of the magnetic kirigami robot. A magnetic sheet without any cut is immobile in plane under a rotating magnetic field. Introducing a U‐cut breaks the symmetry and enables a slow crawling; adding a base cut accelerates the locomotion. B) The crawling happens when opening of the leaf bends the foot and establishes an anchor site. The elastic recovery of the foot during closing of the leaf pushes the robot against the anchor point and generates a propulsive force for crawling. C) Schematics extracted from finite element simulations show the robot with combined U‐cut and base has a foot compared to a robot made with only a U‐cut. D) The mean ± SD values (*n* = 3) for the crawling speed of the robot as a function of the hinge width *δ*. E) Snapshots of a magnetic kirigami robot that crawls in response to a rotating magnetic field. F) Fabrication of magnetic kirigami crawlers by laser cutting a soft magnetic sheet made from a mixture of strontium ferrite powder and a soft silicone rubber, followed by assembly with a stiffer silicone frame.

A plain soft magnetic sheet in which the magnetic dipoles are perpendicular to the thickness beats out of the plane in response to a rotating magnetic field but remains still in the plane (**Figure** **1**A‐i and Movie S1, Supporting Information). Although a rotating field is inherently asymmetric, the resulting interactions are insufficient for friction symmetry breaking and generating propulsion. If we introduce a U‐shaped cut into this magneto‐responsive structure, we create a soft crawling robot with a leaf‐like appendage (Figure 1C, top). The leaf flaps upon application of a simple rotating magnetic field, breaking the friction symmetry and generating propulsive force for crawling (Figure 1E). Adding a base cut to the U‐cut improves the crawling efficiency of the robot. In this case, the leaf has a free end and a flexible base hinged at two extremes that, upon bending the leaf, equip the robot with a foot‐like appendage (Figure 1C, bottom).

A rotating magnetic field deforms the leaf and its foot, enabling the robot to engage with the substrate and crawl (Figure 1A‐iii). Notably, the interaction of the kirigami with the substrate results in different kinetics under a rotating magnetic field during the leaf's and the foot's touching and lifting periods. These interactions generate anisotropic friction and a propulsive thrust in the direction of the motion (Figure 1B). We observed that the crawling speed can be tuned by varying the geometry of the cuts and the width of the hinge separating the leaf from its base (Figure 1D). In addition to the shape of the cuts, the magnetic stimulus can be changed to provide ample design space to control and steer the robot. Next, we systematically study the effect of the geometry of the cuts and the orientation of the magnetic field on the locomotion mechanism of magnetic kirigami robots. The understanding gained is finally exploited to program the complex locomotion behavior of the soft crawling robot.

## 2 Results

### 2.1 Fabrication of Magnetic Kirigami Robots

We fabricated a soft magneto‐responsive thin sheet with a thickness of 1 mm from a composite material obtained by dispersing hard magnetic strontium ferrite (SrFe_12_O_19_) powders in an uncured soft silicone elastomer (Ecoflex 00–20, Smooth‐On). We cast the obtained mixture into a mold and cured it in an oven at 60 °C for 20 min while placing it atop a NdFeB permanent magnet (70 mm × 70 mm × 30 mm, strength: 0.4 T). To align the magnetic particles’ dipoles perpendicular to the sheet thickness, the North Pole (N) face of the magnet is placed in direct contact with the substrate (see Figure 1F). Then, we cut kirigami patterns into the magnetic sheet using a laser cutting machine (Speedy 400, Trotec). To avoid uncontrolled motion of the sheet during magnetic actuation, we glued the magnetic sheet to a rectangular frame (thickness 1 mm) made of stiffer silicone elastomer (Ecoflex 00–20, Smooth‐On, ZA 50 LT, Zhermack, at 30:70 ratio). The flexible frame kept the magnetic sheet flat without compromising compliance (see Fabrication Section in the Experimental Section for more details).

### 2.2 Locomotion Mechanism

Subjecting a flat magnetic sheet to a rotating magnetic field does not result in any noticeable movement because the friction between the robot and the substrate is symmetric (Figure 1A‐i and Movie S1, Supporting Information). To break frictional symmetry, we introduced asymmetric incisions into the magnetic sheet that turned it into a soft crawling robot.

In the first design iteration, we cut a U‐shaped profile with an edge length of *l*, resulting in a leaf‐like appendage that could repeatedly flap under the action of a rotating magnetic field. The leaf is free to move along and follow the rotating external field, as long as it is neither blocked by the underlying substrate nor self‐adhered to the silicone sheet. The blocking effect of the substrate during each cycle and the U‐shape of the cut make the system inherently asymmetric and strongly affected by the direction of the rotation, the deformation of the leaf, and the geometry and history of the contact. To explore this rich parameter space, we first performed an experiment where the magnetic sheet with a U‐cut was subjected to a clockwise (CW) rotating magnetic field and observed a minimal displacement, as shown in Figure 1A‐ii (middle row) and Movie S1 in the Supporting Information. In this and later experiments, we assume that the leaf initially lies flat onto the underlying substrate, and that its tip was pointing toward the right side of the piece of paper used as substrate. Also, we chose the initial configuration of the external field such that the leaf is attracted toward the substrate at the beginning of each cycle. Upon CW rotation of the external magnetic field, the leaf first pushes the substrate due to the magnetic attraction. Beyond a limit point, the interaction between the magnet and the leaf became repulsive, and the leaf sprang back with a catapult motion that moved the robot due to the momentum exchange.

In the next design iteration, we added a line cut with an offset to the base of the leaf, leaving two small ligaments between the leaf's root and the new cut with a distance *δ* (Figure 1A‐iii and Movie S1, Supporting Information). Such base cut allows for the formation of a foot at the base of the leaf (Figure 1C, bottom row). Under the rotating field, the asynchronous motion of the foot and the whole leaf leads to two‐anchor locomotion during the leaf opening and closing phases, respectively. During magnetically induced locomotion, the bending of the leaf deformed the foot into a curved inclined edge that enhanced the friction with the ground (Figure 1B). Upon closing the leaf, the elastic recovery of the sheet straightened the foot and pulled the robot's body toward its anchor point.

Our experiments reveal that the additional line cut led to the formation of a foot that enabled the new robot to establish a longer stride and to crawl faster than the robot with only a U‐cut. We characterized the effect of hinge width *δ* on the speed of crawlers and found that when the offset is very small or very large, the contribution of the foot to the locomotion was minimal. For small *δ*, the anchorage was weak, probably due to the low contact area between the foot and the substrate. For a large *δ*, the foot region was stiff and could not tilt the base and grip the ground. Notably, for medium hinge width, the robot speed increased by multiple folds (Figure 1D). We should consider that varying the size of the base cut and the leaf may also affect the crawling speed (see Figure S1, Supporting Information), however we will not study it here.

### 2.3 Rotational Direction of the Magnetic Field Alters Locomotion Mechanism

**Figure 2:**
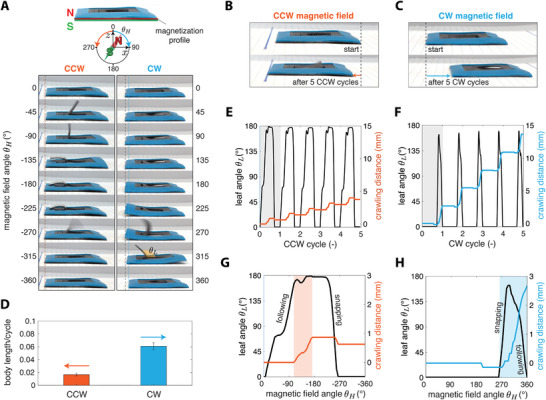
Crawling of the robot in clockwise (CCW) and clockwise (CW) magnetic field. A) Snapshots of the robot during one CCW and CW cycle at different magnetic field angle *θ*_*H*_. Magnet's rotational speed is 1.6 Hz in all experiments. Snapshots of the robot after five cycles moving B) toward the base in CCW magnetic field and C) toward the leaf's tip in CW magnetic field. D) Normalized speed of the robot in CCW and CW fields (*n* = 3). E,F) Crawling distance and evolution of leaf angle *θ*_L_ as a function of magnet's rotation cycle for CCW and CW fields, respectively. G,H) Zoom‐in of the crawling distance and leaf angle *θ*_L_ in one selected cycle as a function of magnetic field angle *θ*_*H*_ for CCW and CW field, respectively. The shaded regions show the intervals in which the robot crawls in the intended direction within each cycle.

We investigated the effect of the rotation direction of the magnet on the crawling response of magnetic kirigami robots (**Figure** **2**A). The rotation of the applied magnetic field is represented by *θ*_*H*_, which is positive for CW rotation and negative for counterclockwise (CCW) rotation. When *θ*_*H*_ = 0°, the North Pole of the external magnet is perpendicular to the robot. We found that CCW magnetic actuation resulted in leftward crawling toward the base of the leaf (Figure 2B), and CW magnetic actuation propelled the robot rightward toward the tip of the leaf (Figure 2C, see Movie S2, Supporting Information). Remarkably, we observed that the rotation direction alters the locomotion mechanism and hence, the crawling speed.

To highlight the difference between the locomotion under CW and CCW actuation, we tracked the walking distance of the robot and correlated it to the opening and closing of the leaf over several cycles. Side view snapshots of the robot during CCW and CW cycles were taken for this experimental analysis (Figure 2A). The walking distance measurements indicated that under CW actuation, the robot has a longer stride length (total displacement in one full turn of the magnetic field) and shorter backlash (displacement against the crawling direction in one cycle) in each step (Figure 2F,H). As a result, the robot crawled almost four times faster with CW actuation than with CCW (Figure 2D). We characterized the leaf angle, *θ*_L_, by measuring the angle between the horizontal surface and a straight line that connected the leaf's tip to its junction point. Characterization of the leaf angle *θ*_L_ indicated a sharp opening and a smooth closing for CW cycle and a smooth rising and a quick closing for CCW (Figure 2G,H). For CCW, the leaf remained open for a more extended period than for CW.

Opening and closing of the leaf dictated the bending of the foot and affected the state of the contact with the substrate. As shown in Figure 2G, in CCW cycle, the leaf immediately followed the field, however, the robot remained still until a magnetic field angle, *θ*_*H*_ ≈ −105° where the leaf was largely open (*θ*_L_ ≈ 165°). Beyond this point, the robot started crawling backward while the field approached *θ*_*H*_ ≈ −180° and the leaf became fully opened (*θ*_L_ → 180°). Finally, the leaf sprang back around *θ*_*H*_ ≈ −225° and the robot retracted 27%, slowing down further the locomotion. Differently, Figure 2H shows that in CW cycle, the robot started crawling forward as early as the leaf snapped to the open state around *θ*_*H*_ ≈ 270° and almost linearly propelled while the leaf smoothly closed by following the field angle up to *θ*_*H*_ = 360°.

The results of these experiments suggest that the bending of the leaf and hence the deformation of the foot had a prominent effect on the robot's locomotion in both CW and CCW regimes. However, tracking the foot's motion in contact with the substrate during crawling is challenging. Therefore, we need to investigate the role of foot deformation further to understand the crawling mechanism of the magnetic kirigami robot. We should emphasize that the locomotion is not simply due to the dragging of the magnet since it occurs discretely, and the robots not only crawled toward the magnet but also traversed over it and moved away from it.

### 2.4 Numerical Simulations

**Figure 3:**
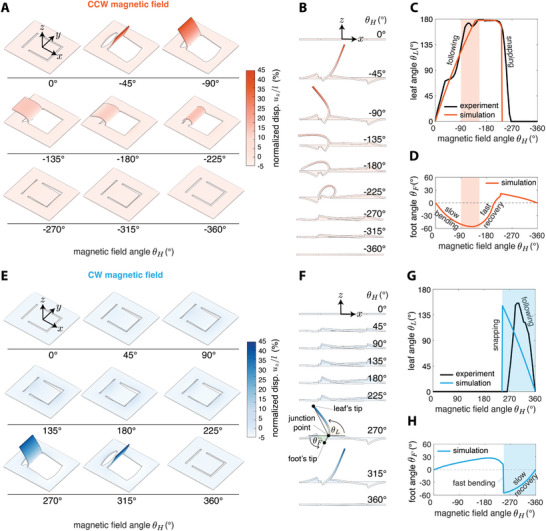
Finite element simulation of magnetic kirigami robot. A,E) Snapshots of the simulated deformation of the robot under CCW and CW rotating uniform magnetic field as a function of the field angle *θ*_*H*_. B,F) Side view snapshots of the robot showing the deformation of the leaf and the base. C,G) Comparing the simulated leaf angle *θ*_L_ with experiments. D,H) Calculated foot angle *θ*_B_ from simulations as a function of the field angle *θ*_*H*_. Color bars show the percentage of the normalized out‐of‐plane displacement *u_z_* with respect to the length of the leaf *l*.

We complemented our experimental work by developing a comprehensive nonlinear finite element model of the magnetic kirigami robot. To create a reliable numerical model, we considered both material and geometric nonlinearities, mechanical instabilities, coupled magneto‐responsive deformation, and contact with the substrate (see the Experimental Section, Modeling for details). We simulated the response of the magnetic sheet under CCW and CW rotating uniform magnetic field. We calculated the displacement field of the robot at different field angles *θ*_*H*_ (**Figure** **3**A,E) from which we estimated the leaf angle *θ*_L_ that despite the highly nonlinear nature of the system agrees very well with experiments (Figure 3C,G). The side view snapshots (Figure 3B,F) of the robot at different field *θ*_*H*_ clearly capture the “following” and “snapping” regimes that were identified in experiments (see Figure 3C,G). This multifaceted agreement between finite element analyses and experiments confirms the validity of the simulation results obtained for these complex multiphysics systems. Hence, we can exploit the results of this model to get deeper insights into the role of the deformation of the foot in the locomotion mechanism of the robot in CW and CCW actuation regimes.

To quantify the deformation of the foot, we defined the foot angle *θ*_F_ by joining the tip of the foot to the junction point of the leaf (see the inset in Figure 3F). The foot angle *θ*_F_ < 0 when the foot is digging into the ground and *θ*_F_ > 0 when the foot is detached from the substrate. The profile of the foot angle *θ*_F_ as a function of the magnetic field angle *θ*_*H*_ shows that for CCW actuation, the base underwent a smooth bending and recovery (Figure 3D), whereas in CW actuation, the bending occurred instantaneously, and recovery happened gradually (Figure 3H). Confronting these observations with crawling profiles (see Figure 3C,G) shows that the crawling in CCW configuration happens mostly at the end of the “bending” phase, whereas in the CW scenario, the robot crawls in the “recovery” phase. These remarkable results suggest that in both actuation protocols, the foot anchors to the ground by bending to *θ*_F_ ≈ −60°. However, in CCW actuation, the foot pushed back the robot against the anchor point whereas in CW actuation, the robot was pulled forward toward the anchor point through elastic recovery. The shaded regions show the intervals in which the robot crawls during each cycle and qualitatively explains why CW actuation is more efficient than CCW one. While in the CW actuation, the whole recovery phase contributes to the locomotion, in the CCW magnetic field, only part of the bending phase participates in locomotion. These behaviors are also clearly visible from top view videos of CCW and CW crawling (see Movie S2, Supporting Information). Additional simulations investigating the deformation of kirigami magnetic robots for different size of the hinge width *δ* under CCW and CW magnetic actuation are shown in Figure S2 in the Supporting Information.

### 2.5 Stick‐Push‐Slip in CCW Actuation versus Slip‐Stick‐Pull in CW Actuation

Locomotion of the magnetic kirigami robot is governed by the deformation‐induced friction asymmetry that is generated by the foot's pedal motion in a rotating magnetic field. The robot can crawl forward and backward by changing the rotation direction of the magnet. The geometry of the kirigami cut creates a leaf‐like appendage that in the presence of substrate makes the dynamics of the system inherently asymmetric in a rotating magnetic field. In contrast to the magnetic field that can rotate continuously, the leaf follows the field partially and sticks to the substrate or folds onto the robot in the rest of the cycle. We found that in CCW actuation, the leaf opens slowly by following the rotating magnetic field and closes with a (fast) snapping instability. Instead, in the CW actuation the situation is reversed: the leaf opens with a quick snapping and closes by following the field. The leaf and the foot form a “magnetic lever” hinged at the junction point that generate a pedal motion on the substrate.

The deformation of the system is controlled by the magnetic forces applied by the external field, the elasticity, and magnetization of the robot. The robot exploits the slow part of the actuation cycle—bending phase in CCW and recovery phase in CW actuation—to stick to the substrate and push (CCW) and pull (CW) itself in the crawling direction. The robot utilizes the fast part of the cycle—recovery phase in CCW and bending phase in CW actuation—to slip the foot over the substrate (without propulsion) and reset the system for the next cycle. The lower speed of CCW actuation compared to CW actuation can be attributed to compression of the foot during bending phase and the fact that only the final stage of the foot's bending contributes to locomotion. In contrast, in the case of CW actuation, the foot is under tension, and the whole period of recovery was spent for crawling. An illustrative comparison for locomotion under CCW and CW actuation is shown in Figure S3 in the Supporting Information. To summarize, we call the locomotion mechanism of CCW actuation as “stick‐push‐slip” where the foot *sticks* to the ground and *push* back the robot and finally *slips* toward the rest position, and name that of CW actuation as “slip‐stick‐pull” where the robot first *slips* to find an anchor point to which it *sticks* and *pulls* the robot toward it.

### 2.6 Programming the Crawling Path

In addition to reversing the crawling direction, the magnetic field can also be used to steer the path of the robot while keeping it under one locomotion mechanism. Alternatively, the locomotion path of the robot can also be programmed within the geometry of the cut. To demonstrate such control and programmability, we exploited the orientation of the rotation axis of the magnet or geometry of the cuts to achieve more complex crawling paths.

**Figure 4:**
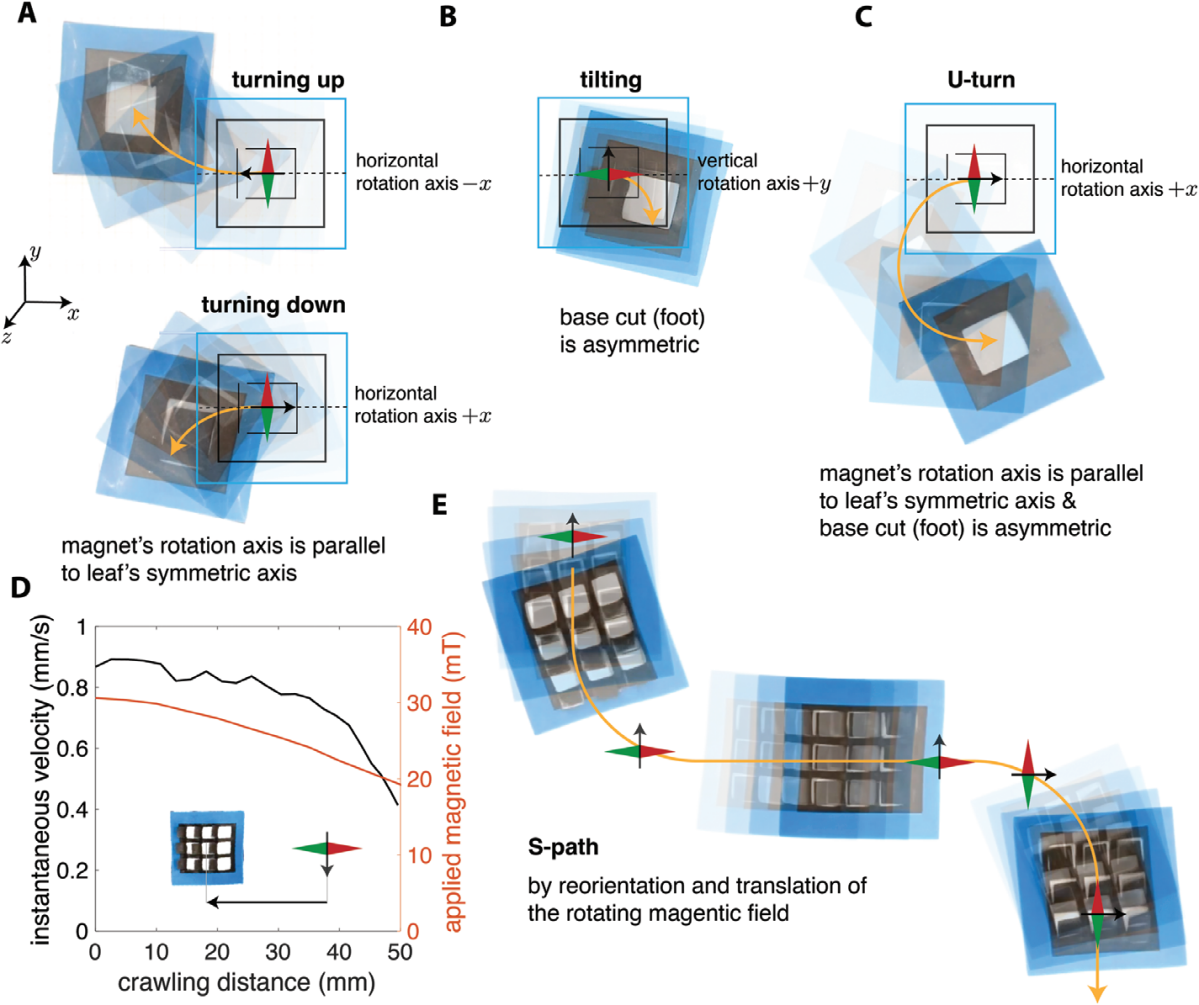
Programming crawling path of magnetic kirigami robots. A) The robot turns up when the magnet's rotation axis is horizontal toward the left and turns down when the rotation axis is toward the right. B) An asymmetric half‐base cut (half‐foot) slightly turns the robot when actuated with a vertical rotating field. C) Combining asymmetric half‐base cut (half‐foot) with a horizontal rotating field results in more significant turning, enabling the robot to make a U‐turn. D) The instantaneous velocity of the robot decreases as it passes over the magnet, and the field weakens with the crawling distance, as shown for the CCW actuation of a magnetic kirigami metamaterial. E) Traversing an S‐shaped path by programming the robot's motion with a sequence of left and right turns combined with the translation of the magnet.

We realized a rotation for a soft magnetic kirigami robot by three methods. First, we applied a magnetic field rotating around the +*x* and −*x* axes (as opposed to previous experiments where the applied field was rotating around the *y*‐axis) to achieve turning in two directions depending on the direction of the rotation. As depicted in **Figure** **4**A, when the applied magnetic field rotated around the −*x* axis, the robot turned upward (around the −*z* axis) and when the rotation was around the +*x* axis, the robot turned downward (around the +*z* axis). The magnetic field rotating around the *x*‐axis breaks the symmetry of the deformation with respect to the horizontal symmetry axis of the robot and the magnetic kirigami crawler turns until the robot aligns itself to become perpendicular to the magnet's rotational axis. In the second method, we introduced a half‐base cut (instead of a full base cut) which resulted in an inherently asymmetric structure that even with a magnetic field rotating around the *y*‐axis showed noticeable tilting (Figure 4B). We also combined these two approaches and activated the robot with the half‐base cut using a magnetic field rotating around the +*x* axis that resulted in a more pronounced turning that can perpetually continue and enable the robot to make a “U‐turn” (Figure 4C).

The high maneuverability of the magnetic kirigami robots is maintained even when the single locomotion units are combined into arrays to create larger crawling metamaterial sheets. The resulting multi‐unit kirigami robot is an untethered soft robot that can be controlled remotely with a rotating magnetic field (Figure 4D,E). However, the performance of the robot declines as the robot gets away from the center of the magnetic field because the magnetic forces weaken. In Figure 4D, we showed this behavior for a magnetic kirigami metamaterial comprised of an array of 3 × 3 U‐cuts under CCW actuation. After crawling about one body length, the strength of the magnetic field felt by the robot decreased by ≈30% and the speed of the robot dropped to half of its maximum speed. We can overcome this limitation by translating the magnet at the same velocity of the crawling robot.

To demonstrate the path‐programmability of a soft crawler based on a magnetic kirigami metamaterial, we used a combination of translational motion and reorientation of the magnet's axis of rotation. The periodic nature of the magnetic kirigami metamaterial and the presence of a gradient in the field strength allow us to intentionally activate selected regions of the soft robot to facilitate maneuvering. In particular, the units that are closer to the external magnet respond more strongly than those that are farther, enabling easier rotation. We performed an experiment where we changed the orientation of the magnet's axis of rotation and translated the magnet in the plane to follow an “S‐path” over a larger area. Figure 4E shows that robot successfully completes the designated path with a few manipulations along the way that included changing the rotation axis from vertical to horizontal and accompanying it with linear translations in three segments (see Movie S4, Supporting Information).

## 3 Conclusion

In summary, introducing kirigami into soft magnetic sheets results in untethered soft crawlers that can locomote with a simple rotating magnetic field. By using a kirigami design consisting of a simple U‐shape and a line cut into a magneto‐responsive sheet, we were able to create soft crawlers with rich locomotion behavior when subjected to a dynamic magnetic field. We exploited this design to achieve crawling in forward and backward directions with CW and CCW rotation of a permanent magnet. We correlated the deformation history of the leaf and the base cut with the crawling profile of the robot to extract a physics‐based interpretation of the crawling mechanism of the robot. Multiphysics finite element simulations of the robot revealed that the deformation of the foot plays a crucial role in the crawling behavior of the robot. These results identified two mechanisms for CCW and CW actuation protocols that we referred to as “stick‐push‐slip” and “slip‐stick‐pull” locomotion. Finally, we manipulated the geometry of the base cut and orientation of the magnetic field to generate turning motion and combined it with the translation of the external magnetic field to program arbitrary planar crawling paths. The kirigami soft robots provide a wide design space for further programmability and control by tuning several other parameters, including the kirigami pattern, stiffness, and magnetization profile. With the implementation of an automated magnetic field control system, we envision the creation of untethered soft robots capable of multimodal complex locomotion. Kirigami magnetic robots that can traverse unstructured terrains and navigate narrow passages inaccessible to traditional rigid robots may find applications in environmental monitoring, space exploration, medical inspection, and robotic surgery.

## 4 Experimental Section

### Fabrication

Soft magnetic sheets were made by mixing the following ingredients: 4 g Ecoflex 00–20 (Smooth‐On), 4 g SrFe_12_O_19_, and 50 mg silicone oil, resulting in a composite with about 50% mass fraction of magnetic particles. The magnetic material was SrFe_12_O_19_ powder (UF‐S2, DOWA Electronics Materials Co. Ltd.) and it had a bimodal particle size distribution of 0.8 and 3 µm. SrFe_12_O_19_ was a hard magnetic material (high magnetic remanence) and was therefore capable to provide a strong permanent magnetic dipole moment once it was magnetized. The polymer into which the SrFe_12_O_19_ particles were mixed was a soft silicone rubber (Ecoflex 00–20, Smooth‐On‐Inc.) with a low 100% elastic modulus of 55 kPa and a high elongation at break of 845%. Silicone oil (Sigma Aldrich) was additionally added to reduce coagulation and improve mixing. The mixture was homogenized and degassed with a centrifugal mixer (ARE‐250 CE, THINKY) and 5 g was poured onto a 2 mm thick polymethylmethacrylate (PMMA) plate. To produce a sheet with a uniform thickness of 0.5 mm, the mixture was pressed together with another PMMA plate and placed on a permanent NdFeB magnet. Optical microscopy and scanning electron microscope images of the prepared composite material with an equivalent mass fraction showed the oriented magnetic particles dispersed uniformly within the matrix (see Figure S4, Supporting Information). The magnetic composite material exhibited a remanent magnetization of 55 emu g^−1^ when magnetic fields between − 0.3 and 0.3 mT were applied. A typical magnetized film used for robot fabrication exhibited remanent flux densities up to 5 mT. A comprehensive characterization of the soft magnetic composite material can be found elsewhere.^[^ ^37^ ^]^

The flexible blue frame was fabricated by mixing the Ecoflex 00–20 with a stiffer silicone rubber (ZA 50 LT, Zhermack SpA) in 30:70 ratio, respectively. ZA 50 LT had a Hardness Shore A50 and an elongation at break of 200%. Without the ZA 50 LT, the frame would be too flexible resulting in an uncontrollable bending of the magnetic sheet in proximity of the rotating magnetic field. The elastomer sheets were laser cut with a 100 W CO_2_ laser (Speedy 400, Trotec). The cutting power was set to 30% with a speed of 80% and 5000 Hz PPI (pulses per inch).

### Actuation

In all the experiments, rotating magnetic fields for actuation were generated by rotating a permanent rare earth NdFeB magnet (50 mm × 40 mm × 20 mm) with a frequency of 1.6 Hz using a DC motor. The distance of the magnet to the specimen was 7 cm, generating a magnetic field magnitude of 10–40 mT at the surface of the magnetic sheet. Also, the effect of the rotating magnetic field's frequency on the crawling speed of a kirigami magnetic robot with 3 × 3 cuts was experimentally investigated and the results are reported in Figure S5 in the Supporting Information.

### Modeling

The magnetic sheet was modeled with a hard‐magnetic soft material model.^[^ ^38^, ^39^ ^]^ The total Helmholtz free energy function (Ω) of the ideal hard‐magnetic soft material per unit volume in the reference configuration could be expressed as

(1) $$\Omega \;\left(F\right)=\Omega_{E}\;\left(F\right)-\frac{1}{\mu_{o}}Fb^{r}\cdot b^{a}$$

where Ω_E_ is the elastic part of energy function and **F** is the deformation gradient tensor. In this equation, **b** ^r^ is the residual magnetic flux density in a hard‐magnetic soft material and **b** ^a^ is the applied magnetic flux density vectors. **b** ^r^ and **b** ^*a*^ both were assumed to be constant values at each location of interest. Therefore, Ω becomes purely a function of **F** and the first Piola–Kirchoff stress tensor could be expressed as (⊗ is dyadic product)

(2) $$P\;\left(F,b^{a}\right)=\frac{\partial \Omega \left(F\right)}{\partial F}\;=\frac{\partial \Omega_{E}\left(F\right)}{\partial F}\;-\frac{1}{\mu_{o}}b^{a}⊗b^{r}$$

Guided by tensile experiments, the behavior of the magnetic sheet was approximated with an incompressible isotropic Yeoh hyperelastic material model. The strain energy density function of this material model was expressed as a function of the first invariants of the right Cauchy–Green deformation tensor *I*_1_ =  tr(*C*)

(3) $$\Omega_{E}\;\left(F\right)=c_{1}\;\left(I_{1}-3\right)+c_{2}{\left(I_{1}-3\right)}^{2}+c_{3}{\left(I_{1}-3\right)}^{3}$$

where *c*_1_ =  11 kPa, *c*_2_ =  5.5 Pa, and *c*_3_ =  0.55 Pa are material constants identified by fitting the force–displacement curve to tensile experimental data. The initial shear modulus of the material is estimated as *G*  =  22 kPa.

The residual magnetic flux density **b** ^r^, and applied magnetic flux density vector **b** ^a^, were prescribed as follows

(4) $$\frac{1}{\mu_{o}}b^{a}⊗\;b^{r}=\;bP_{M}\left(\theta \right)\;$$

(5) $$P_{M}\;\left(\theta \right)=\left[\begin{array}{c} \cos\theta \\ \sin\theta \\ 0 \end{array}\right]\;⊗{\bar{b}}^{r}$$

in which *θ* defines the direction of the applied magnetic flux density, ${\bar{b}}^{r}$ is the residual magnetic flux density unit vector obtained from the direction of magnetic dipoles within the material, and **b**  =  110 Pa which measures the strength of the magnetic interaction and was calibrated with the experimental deformation of the magnetic sheets. Therefore, the first Piola–Kirchoff stress tensor as a function of the deformation gradient **F**, the magnetic interaction parameter **b**, and the rotating magnetic field angle *θ* was given by

(6) $$P\;\left(F,b,\theta \right)=\frac{\partial \Omega_{E}\left(F\right)}{\partial F}\;-bP_{M}\left(\theta \right)$$

The geometry of the robot was discretized and the displacement field variables with a 15‐node quadratic 3D solid element were interpolated. The governing equations of motion were extracted by implementing the Hamiltonian principle and considering the above‐mentioned nonlinear constitutive model under finite strain regime. The resulting nonlinear system of equations was numerically solved with Newton–Raphson method using an in‐house code developed in MATLAB. In the solution process, the Riks technique^[^ ^40^ ^]^ was employed to trace nonlinear deformations caused by the rotation of the magnetic field.

### Statistical Analysis

In Figures 1D and 2D, crawling speed of the robot data was acquired by averaging a minimum of three measurements (*n* = 3) and the data were reported as mean± SD values.

## Conflict of Interest

The authors declare no conflict of interest.

## Supporting information

Supporting Information

Supplemental Movie 1

Supplemental Movie 2

Supplemental Movie 3

Supplemental Movie 4

## Data Availability

The data that support the findings of this study are available from the corresponding author upon reasonable request.
