# Supplementary material for: Kirigami Makes a Soft Magnetic Sheet Crawl
Source: Adv Sci (Weinh). 2023 Jun 25;10(25):2301895. doi: 10.1002/advs.202301895 (PMC10477847; doi:10.1002/advs.202301895)
Supplement: Supplementary file 1 — Supporting Information [file ADVS-10-2301895-s004.pdf]

ADVANCED SCIENCE
Open Access

Supporting Information

for *Adv. Sci.*, DOI 10.1002/advs.202301895

Kirigami Makes a Soft Magnetic Sheet Crawl

Pierre Duhr, Yuki A. Meier, Alireza Damanpack, Julia Carpenter, André R. Studart, Ahmad Rafsanjani and Ahmet F. Demirörs**

Supporting Materials

Kirigami makes a soft magnetic sheet crawl

Pierre Duhr[1§], Yuki A. Meier[1§], Alireza Damanpack[2§], Julia Carpenter[1], André R. Studart[1] Ahmad Rafsanjani[*,3], and Ahmet F. Demirörs[*,1]

[1]Complex Materials, Department of Materials, ETH Zurich, 8093 Zurich, Switzerland

[2]Department of Mechanical and Electrical Engineering, University of Southern Denmark, 5230 Odense, Denmark

[3]SDU Soft Robotics, SDU Biorobotics, The Maersk Mc-Kinney Moller Institute, University of Southern Denmark, 5230 Odense, Denmark

§ These authors equally contributed to this work.

*Corresponding authors

E-Mails: ahra@sdu.dk, ahmet.demiroers@mat.ethz.ch

Date: May 28, 2023

Effect of varying the geometrical parameters of kirigami on maximum achievable velocity

We experimentally characterized the effect of varying geometrical parameters of magnetic kirigami sheets on the maximum achievable velocity of the robot compared to a reference design with $l = 14$ mm, $w = 45$ mm, $t = 7.5$ mm, and $\delta = 3.5$ mm. Three considered cases are:

- Small foot: by decreasing the length of the base cut by 20%.
- Big foot: by increasing the length of the base cut by 20%.
- Small leaf: by downscaling the size of the leaf by 20%.

The maximum velocity of the robot decreased in all considered cases compared to the reference design as shown in Figure S1.

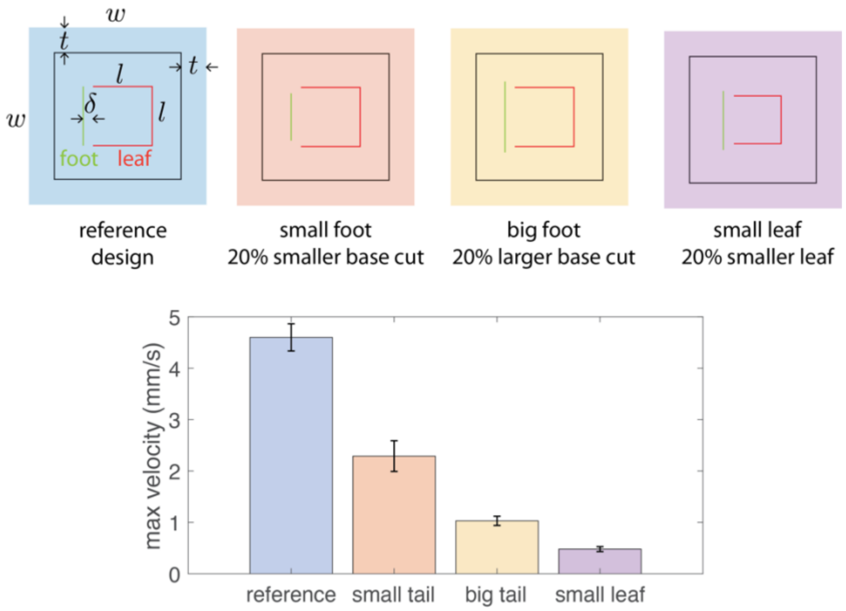

Figure S1. Effect of varying the size of the foot and the leaf on maximum velocity (n=3) of the robot compared to a reference design with $l = 14$ mm, $w = 45$ mm, $t = 7.5$ mm, and $\delta = 3.5$ mm.

Effect of hinge width on foot angle for CCW and CW magnetic fields

Finite element simulations of magnetic kirigami robots for different values of hinge width δ under CCW and CW magnetic actuation.

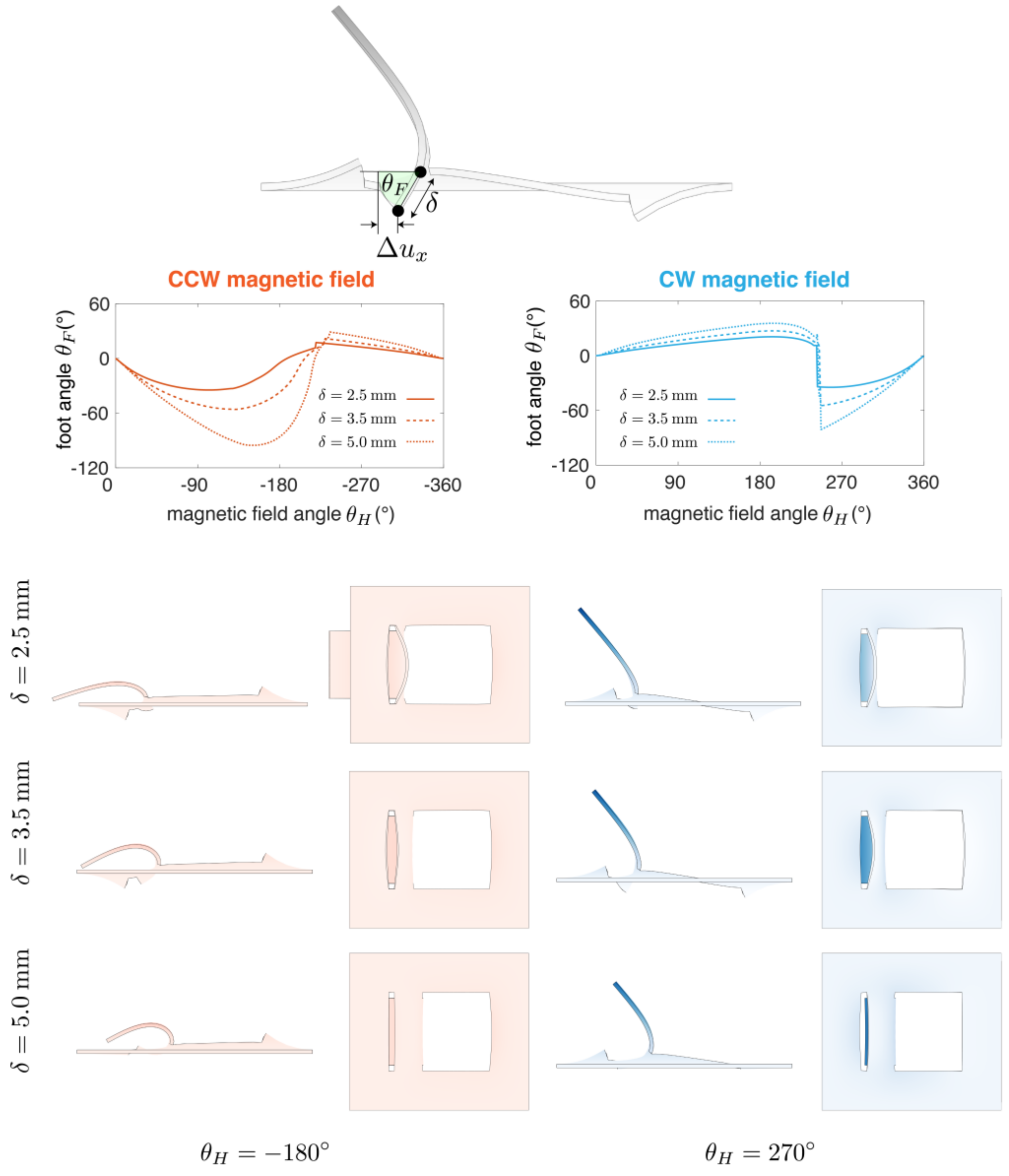

Figure S2. Evolution of the foot angle θ_F of kirigami magnetic robots for different values of hinge width δ under CCW and CW magnetic actuation. Snapshots of the deformation of the kirigami magnetic robots at selected angles θ_H of the rotating magnetic field.

Comparing the locomotion mechanisms under CCW and CW actuation

Our analyses suggest that the speed difference is attributed to how the foot deforms, anchors to the ground, and relaxes. We note that under CCW actuation, the locomotion happens while the foot is under compression by friction force, and only the last stages of the bending of the foot contribute to crawling. On the other hand, under CW actuation, the foot is under tension, and the full extent of the relaxation of the deformed foot contributes to propulsion. Hence, the CW actuation is faster than CCW. We illustrated the difference between crawling behavior under CW and CCW actuation in Fig. S3.

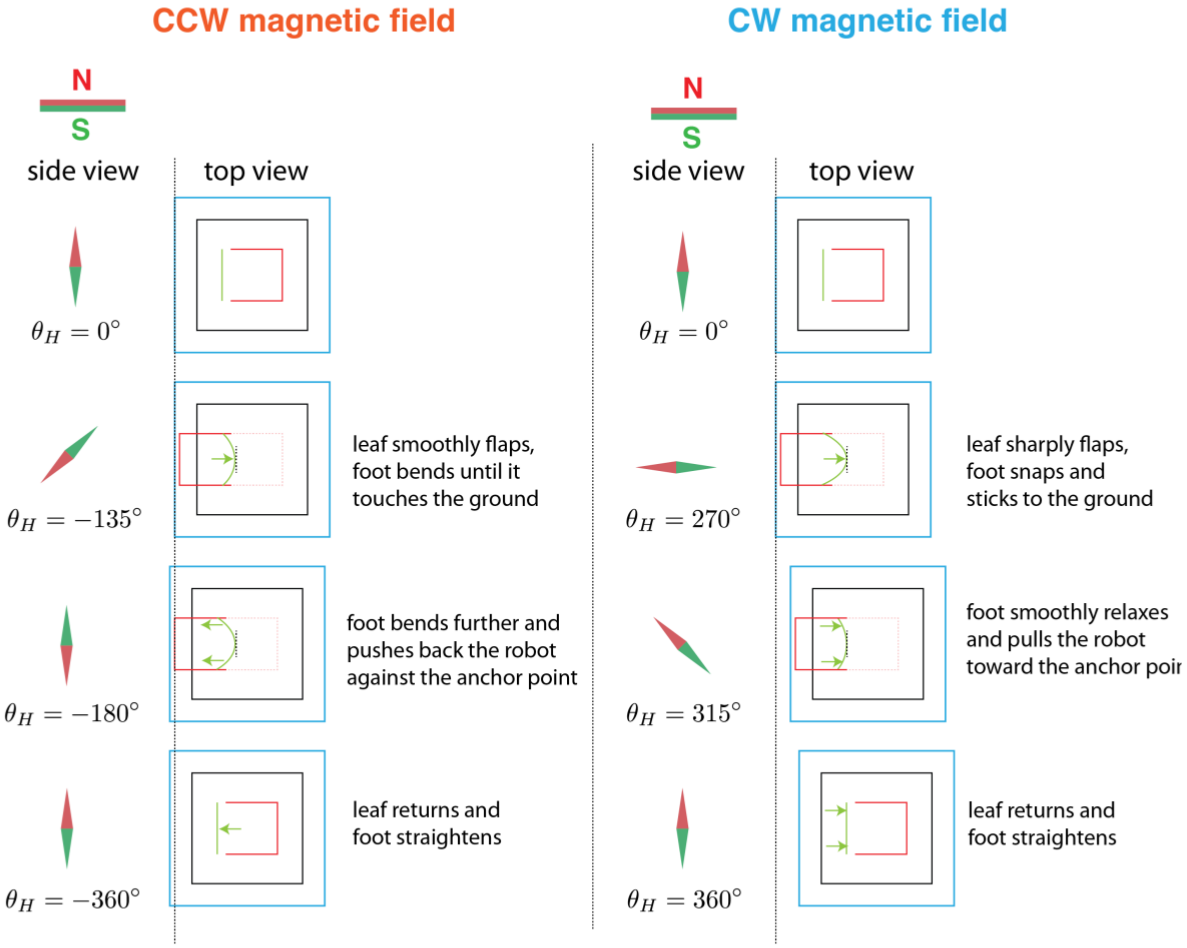

Figure S3. Comparison between the mechanisms of locomotion under CCW and CW magnetic fields.

Distribution of magnetic particles in the silicone matrix

The distribution of the magnetic particles within the silicone matrix is very homogeneous as shown in Fig. S4. Optical microscopy and SEM images of the prepared composite material with an equivalent mass fraction show the oriented magnetic particles dispersed uniformly within the matrix.

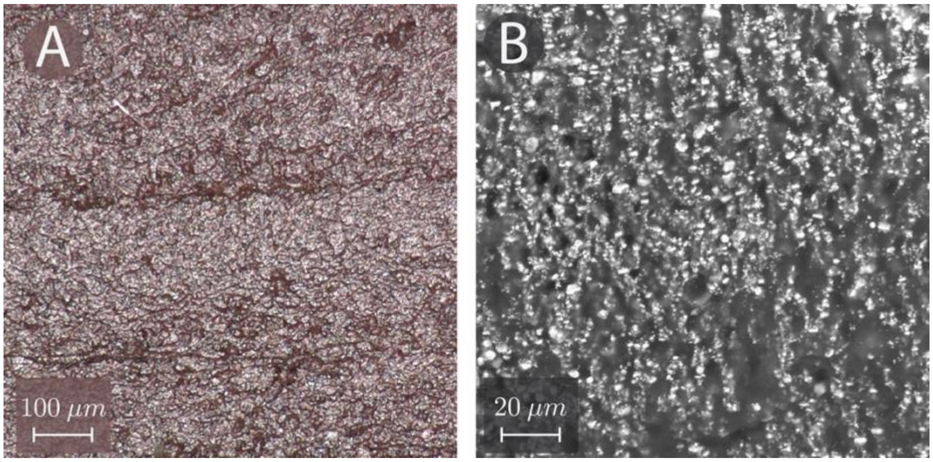

Figure S4. (A) optical microscopy and (B) scanning electron microscopy (SEM) images through the thickness of a composite prepared with Strontium Ferrite magnetic particles.

Effect of frequency of rotating magnetic field on robot's velocity

We experimentally analyzed the correlation between crawling speed and rotation frequency of the magnetic field within the operational limits of our experimental setup. This investigation focused on a sample featuring 3×3 U-cuts, but we anticipate that this relationship also holds for other designs. Our examination involved progressively increasing the frequency of a clockwise rotating magnetic field from 1.6 Hz to 4.2 Hz. However, excessive vibrations in our setup hindered accurate assessment of the robot's locomotion at frequencies beyond this range.

Our findings revealed that as the frequency of the rotating magnetic field increased, the robot's crawling speed also accelerated. Nevertheless, we anticipate that the inertial effect will prevail beyond a critical frequency, causing a sharp decline in speed as the robot's leaf cannot effectively follow the magnetic field. Fig. S5A and S5B, respectively, show the evolution of the robot's velocity with the walking distance and the maximum velocity as a function of the frequency of the rotating magnetic field. In Fig S5A, we observe that the robot speed increases as the robot crawls over the magnet and declines when it takes distance from it due to a weaker magnetic field. In Fig. S5B, we notice that the gain in the speed by increasing the frequency is at a lower rate for higher frequencies.

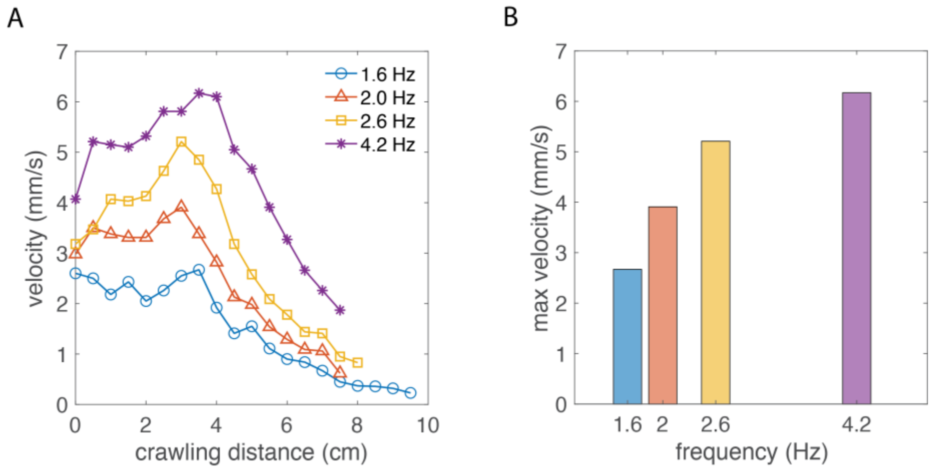

Figure S5. (A) Evolution of the robot's velocity with crawling distance, and (B) maximum velocity for different frequencies of the rotating magnetic field. The experiments were performed for a magnetic kirigami robot with 3×3 U-cuts. In this frequency range, the speed increases with increasing frequency. We could not go beyond 4.2 Hz due to the limitations of the setup.

Description of Supplementary Movies

Movie S1. Comparing the crawling of three magnetic sheets without cuts, with U-cut, and with U-cut + base cut.

Movie S2. Crawling of a kirigami magnetic sheet under CW and CCW rotating magnetic fields.

Movie S3. Finite element simulations of kirigami magnetic sheets with and without base cuts under CW and CCW rotating magnetic fields.

Movie S4. Demonstrating different strategies for programming the crawling path of kirigami magnetic sheets.
